# Association between Motives for Dish Choices during Home Meal Preparation and Weight Status in the NutriNet-Santé Study

**DOI:** 10.3390/nu8070413

**Published:** 2016-07-05

**Authors:** Pauline Ducrot, Philippine Fassier, Caroline Méjean, Benjamin Allès, Serge Hercberg, Sandrine Péneau

**Affiliations:** 1 Université Paris 13, Equipe de Recherche en Epidémiologie Nutritionnelle, Centre de Recherche en Epidémiologie et Statistiques, Inserm (U1153), Inra (U1125), Cnam, COMUE Sorbonne Paris Cité, Bobigny F-93017, France; p.fassier@eren.smbh.univ-paris13.fr (P.F.); c.mejean@eren.smbh.univ-paris13.fr (C.M.); b.alles@eren.smbh.univ-paris13.fr (B.A.); s.hercberg@eren.smbh.univ-paris13.fr (S.H.); s.peneau@eren.smbh.univ-paris13.fr (S.P.); 2 Département de Santé Publique, Hôpital Avicenne, Bobigny Cedex F-93017, France

**Keywords:** dish choices, home meal preparation, body mass index, cross-sectional study

## Abstract

Although home cooking has been associated with a lower body mass index in a few studies, no data exists on the motives behind food dish choices during home meal preparation and on their association with overweight. This study aimed to evaluate this association in 50,003 participants from the NutriNet-Santé cohort. Dimensions underlying the importance of 27 criteria possibly influencing dish choices were determined using an exploratory factor analysis. The association between dish choice motives and overweight (including obesity) was estimated using logistic regression models adjusted for sociodemographic and lifestyle characteristics. Five dimensions of dishes choice motives emerged: healthy diet (e.g., “nutritional balance of the dish”), constraints (e.g., “my cooking skills”), pleasure (e.g., “originality of the dish”), specific diets (e.g., “my health status”), and organization (e.g., “what I planned to eat”). A negative association was observed between the healthy diet factor and being overweight (OR = 0.65 (95% CI (confidence interval): 0.62–0.67)), whereas a positive association appeared for factors regarding pleasure (OR = 1.14 (95% CI: 1.10–1.19)) and specific diets (OR = 1.19 (95% CI: 1.17–1.22)). No significant associations were observed for constraints and organization. The significant associations between dish choice motives and overweight suggested the interest of focusing on these motives in order to promote healthier food choices during home cooking.

## 1. Introduction

In 2013, more than one third of the world population was overweight or obese [1]. Given the established health risk related to weight gain, obesity has become a global health challenge [2]. In western societies, the modification of lifestyles, such as women’s increased role in the paid work force [3] and the expansion of available food options [4,5,6] resulted in the evolution of cooking practices [3,7,8], which may have influenced dietary behaviors [8,9,10,11] and consequently weight status [11,12,13].

Over the last few decades, the time devoted to home-meal preparation has strongly decreased [8] and people have more frequently turned towards relying food prepared away from home, including foods from restaurants, fast-food, take-out, delivery, or ready prepared meals. Compared with home prepared food, food prepared away from home tend to contain more total fat and saturated fat on a per-calorie basis [10,14,15], potentially explaining why a higher consumption of such foods has been linked with higher body mass index (BMI; calculated as kg/m^2^) [12,13,16]. In contrast, more frequent home food preparation has been associated with a lower reliance on away from home food (i.e., fast-food and fried foods) [17], a better adherence to dietary guidelines [17] and higher intakes of healthy foods [18,19]. Additionally, an increased amount of time spent on cooking has been linked to a lower BMI [11,20]. Furthermore, home food preparation might be a means in which to increase the frequency of family meals, which has previously been associated with lower risk of child overweight/obesity [21,22,23,24,25,26].

Given that home food preparation may have a positive impact on body weight, it seems crucial to better understand the motives influencing these food choices and their potential relationship with body weight. Consumers’ reasons for choosing ready-meals have been fully described in the literature. The main motivations generally refer to the convenience and value for money compared to home cooking [15,27,28,29,30]. Moreover, previous studies have evaluated motivations and barriers for home cooking, and their relationship with weight status. In particular, time constraints and cooking skills have been identified as major barriers for home cooking [3,7,13,17,31] and were shown to be positively related with overweight [13,20,32].

In turn, only one previous study fully explored the motivations behind dish choices for home meal preparation [33]. It showed that a number of motives related to constraints, pleasure, organization, and health related aspects, are likely to influence dish choices at home. However, there is no data in the literature regarding the association between motives for food prepared at home and weight status.

Therefore, the purpose of the present study was to explore the association between the motives behind dish choices during home meal preparation and overweight (including obesity) in a large sample of French adults.

## 2. Materials and Methods

### 2.1. Study Population

NutriNet-Santé [34] is an ongoing web-based prospective observational cohort study launched in France in May 2009 with a scheduled follow-up of 10 years. It aims to investigate the relationship between nutrition and chronic disease risk, as well as the determinants of dietary behavior and nutritional status. The study was implemented in the general French population (internet-using adult volunteers, age ≥18 years). The rationale, design, and methodology of the study have been fully described elsewhere [35]. In brief, to be included in the study, participants complete a baseline set of self-administered, web-based questionnaires assessing dietary intake, physical activity, anthropometric characteristics, lifestyle, socioeconomic conditions, and health status. As part of the follow-up, participants are requested to complete the same set of questionnaires every year. Moreover, each month, participants are invited by e-mail to fill in optional questionnaires related to dietary intakes, determinants of eating behaviors, and nutritional and health status. This study is conducted in accordance with the Declaration of Helsinki, and all procedures were approved by the Institutional Review Board of the French Institute for Health and Medical Research (IRB Inserm No. 0000388FWA00005831) and the Commission Nationale de l’Informatique et des Libertés (CNIL No. 908450 and No. 909216). All participants provided informed consent with an electronic signature. This study is registered in EudraCT (No. 2013-000929-31).

### 2.2. Data Collection

#### 2.2.1. Dish Choice Questionnaire

Data concerning dish choices were collected in September 2013 via an optional questionnaire, accessible on the secured personal account of the participants for a six month period. Information as to whether the participant was involved in the choice of dishes for home meal preparation was collected (never, sometimes, often, always) and participants who responded “never” were further exempted to complete the questionnaire. The questionnaire assessing determinants of dish choices was generated based on existing literature and the expertise of nutritionists, epidemiologists, sociologists, and sensory specialists. The questionnaire included 27 items on dish choice motives, including commonly recognized factors such as preferences, eating habits, cooking practices, health, and constraints related to time and food availability. Participants were asked the following question: “When choosing the dishes you are going to cook, how important are the following criteria?” The responses were rated on a 5-point Likert scale ranging from 1 (not important at all) to 5 (very important), with each point on the scale represented by a word anchor. Information about dish choice motives was collected for weekdays and weekends separately.

The full questionnaire is available elsewhere [33].

#### 2.2.2. Anthropometric Measures

Height and weight were assessed by using an anthropometric questionnaire, which was self-administered online, at baseline and each year thereafter [36,37]. For each participant, the closest available data to the dish choice questionnaire were used for the analysis.

Data were not collected for pregnant women. BMI (in kg/m^2^) was calculated as the ratio of weight to squared height. Participants were classified as underweight or normal weight (BMI < 25) and overweight (including obesity; BMI ≥ 25) according to World Health Organization reference values [2].

#### 2.2.3. Sociodemographic and Lifestyle Data

At baseline and annually thereafter, participants in the NutriNet-Santé study are asked to provide socio-demographic data including sex, age (18–29, 30–49, 50–64, ≥65), educational level (up to secondary, some college or university), household status (living alone without children, living with a partner without children, household with at least one child, household with at least one adolescent, household with children and adolescents), dieting in the past year (yes, no), and monthly income (<1200, 1200–1799, 1800–2699, ≥2700 per consumption unit). Monthly household income was calculated per “consumption unit” (CU), where one CU is attributed for the first adult in the household, 0.5 CU for other individuals aged 14 or older, and 0.3 CU for children under 14, following national statistics methodology and guidelines [38]. Physical activity level (low, moderate, high) was assessed using a short form of the French version of the International Physical Activity Questionnaire (IPAQ). The weekly energy expenditure expressed in metabolic equivalent task minutes per week was estimated, and three scores of physical activity were constituted (i.e., low (<30 min/day), moderate (30–59 min/day), and high (≥60 min/day)) according to the French guidelines for physical activity [39].

For each participant, the closest available data to the dish choice questionnaire were used for the analysis.

### 2.3. Statistical Analyses

Student’s *t*-tests and chi-square tests were used to compare included and excluded participants (i.e., participants not involved in dish choice, pregnant women, and individuals with missing data on the outcome), as well as overweight and non-overweight participants, as appropriate. Dish choice motives were identified in a previous study, fully described elsewhere [33] using an exploratory factor analysis conducted on the 27 items related to dish choices. From this work, five dimensions emerged (i.e., healthy diet, constraints, pleasure, specific diets, and organization) and mean ratings were calculated based on loading criteria. Mean scores ranged from 1 to 5 points. A multivariate logistic regression analysis, including all dish choice motives and covariates, was performed to compare the associations between the five dish choice motives and overweight. To evaluate whether some items weighted more specifically within certain associations and because some items might exhibit differential associations within certain factors (e.g., vegetarian diets vs. diet to lose weight), secondary analyses between the 27 dish choice motive items and overweight were performed using multivariate logistic regression analyses. Potential covariates were identified based on evidence in the literature: sex [40], age, educational level [41], income [42], family status, history of dieting, and physical activity [43]. Confounders that reached *p* < 0.15 in univariate models were further combined in a multivariate model. Variables reaching *p* < 0.05 were further retained in the final multivariable model which included sex, age, educational level, income, family status, history of dieting, physical activity, and other dish choice motives (e.g., the “healthy diet” model was adjusted for the four other dish choice motives). History of dieting was not considered in the association between meal planning and the “eventual diet to lose weight” item, given the potential collinearity. Missing covariate data for educational level, monthly income/CU, and physical activity were imputed using the multiple imputation method.

Significant interactions were observed between sex and two dish choice motives (constraints (*p* = 0.01) and pleasure (*p* = 0.007)). However, analyses stratified by sex showed very similar results. All results were therefore presented for men and women together.

All tests of significance were two-sided, and a *p*-value < 0.05 was considered significant. Statistical analyses were performed with SAS software (version 9.4; SAS Institute Inc., Cary, NC, USA).

## 3. Results

### 3.1. Characteristic of the Sample

A total of 53,025 participants answered the questionnaire about dish choice motives (i.e., 33.8% of volunteers who were invited to complete the questionnaire on the NutriNet-Santé website [34]). Among this sample, we excluded 2110 participants who reported never being involved in dish choice, 722 pregnant women, and an additional 190 participants because of missing data for height and weight. Our final sample thus included 50,003 participants.

Compared with excluded participants, included participants were more often women (*p* < 0.0001), were younger (*p* < 0.0001), had a higher educational level (*p* < 0.004), were more likely to live alone and to have children living at home (*p* < 0.0001), were more physically active (*p* < 0.0001), and were less likely to have been on diet to lose weight over the past year (*p* < 0.0001). 

Characteristics of the studied population are presented in Table 1. Compared with non-overweight individuals, participants of the overweight group were more often men, were older, had lower income and educational levels, were more likely to live in a household without children, were less physically active, and were more likely to have been on a diet to lose weight over the past year (all *p* < 0.0001).

### 3.2. Exploratory Factors Analysis

Results of the exploratory factor analysis have been fully described elsewhere [33]. A five-factor solution emerged from the exploratory factor analysis. The first factor explained 48.3% of the total variance and consisted of five items corresponding to healthy eating motives. The second factor accounted for 19.0% of the total variance and included six items, all referring to constraints. The third factor accounted for 12.1% of the total variance and comprised five items referring to pleasure. The fourth factor explained 11.0% of the total variance and consisted of three items related to specific diets. Finally, the fifth factor accounted for 9.6% of the total variance and included three items concerning meal organization. Table 2 shows mean ratings displaying the importance attached to all of the dish choice motives in overweight and non-overweight groups. Overall, only few differences were observed between the two groups. The non-overweight group showed higher scores on the healthy diet and constraints factors, but lower scores for pleasure and specific diets factors, compared with the overweight group. No significant difference was observed for organization and all items connected to this factor. In terms of hierarchy, in both overweight and non-overweight groups, having a healthy diet was the most important motive, followed by constraints and pleasure. In turn, specific diets motive was more important than organization in overweight individuals while the opposite was observed in non-overweight group. The main difference was found for the importance attached to the criteria “My eventual diet to lose weight and/or that of my relatives”, which was higher in overweight group.

### 3.3. Association between Dish Choice Motives and Overweight

Results showing the associations between dish choice motives and overweight are presented in Table 3. Since similar associations were observed in both contexts (i.e., weekdays, weekend) and because weekdays represent a larger part of the diet, only results for this context were presented. The healthy diet was negatively associated with overweight and showed the strongest association while the pleasure and specific diets factors were positively associated. No significant association was found for the constraints and organization factors.

Table 4 shows the associations between the 27 criteria of dish choice motives and overweight. All criteria related to the healthy diet factor were negatively associated with overweight. Within the constraints factor, the items “time available for cooking” and “My hunger and/or that of my relatives” showed a slight negative association whereas “my state of fatigue” was positively associated with overweight. All the criteria connected to the pleasure factor exhibited a slight positive association with overweight. The three items belonging to the specific diet factor were associated with overweight, but “my eventual diet to lose weight and/or that of my relatives” and “my health status and/or that of my relatives” were associated positively while “my personal convictions and/or that of my relatives” showed a negative association. Within the organization factor, only the item “The dish can be prepared beforehand” appeared slightly negatively associated with overweight.

## 4. Discussion

Our results highlighted that specific dish choice motives during home meal preparation are significantly associated with overweight. The healthy diet factor was negatively associated with overweight, and showed the strongest association while specific diet and pleasure factors were positively associated with overweight. No significant associations were observed for constraints and organization factors. However, significant associations could be found for specific items constitutive of these factors.

To date, studies have mainly focused on the nutritional quality of food prepared at home [10,17,18] and away from home [10,12,13,44,45,46]. While more frequent food preparation at home was associated with a healthier diet [17,18], a higher consumption of food prepared away from home was associated with lower dietary quality [45,46] and higher weight status [12,13,45], thus suggesting the potential beneficial impact of home cooking. Our data bring new insights to the literature by showing the importance of the choices made at home when preparing meals. Our data specifically showed that a number of motivations behind dish choices are associated with a higher weight status. One potential explanation is that the motives under dish choice may result in more or less healthful options [28], thus impacting diet quality and weight status.

In the present study, individuals who gave importance to a healthy diet when choosing the dishes for meal preparation were less likely to be overweight. To our knowledge, this study is the first to evaluate the potential relationship between motives behind dish choices and overweight. In the literature, some studies have investigated the association between food choice motives and dietary quality [47,48,49,50], but they were generally performed on specific populations (e.g., women, parents) and evaluated the consumption of particular food groups such as fruits and vegetables. Overall, despite these differences, interest in healthy eating has been associated with better dietary quality [47,48,50], thus potentially explaining the negative association observed between motives and overweight in our sample. However, to our knowledge, only one study evaluated the relationship between interest in healthy eating and BMI, and found no significant association [50]. This discrepancy might be due to the fact that this study was performed on parents only while our study was based on the general population. Although interest in a healthy diet might prevent the occurrence of overweight, the reverse causality cannot be excluded since interest on healthy diet could be due to non-health related reasons, such as concern about appearance and weight [51,52]. In line with this idea, in Hispanic and African American women caretakers, normal weight women have been shown to be more likely to place importance on consuming healthy food, compared with overweight caretakers [53]. Secondary analyses showed that “nutritional balance of the dish”, “nutritional balance of the meal”, and “what I and/or my relatives ate during the previous days” items exhibited a negative association with overweight, which is consistent with the fact that diet balance is a key factor for weight maintenance either on the short or on the long term [54,55,56].

In our study, we found no significant association between the constraints factor and overweight. This result diverges from data of previous literature which suggested that constraints related to home meal preparation, such as time scarcity or low cooking skills, might increase the use of convenience food [13,57], and finally lead to weight gain [13,20]. Additionally, when considering items constitutive of the factor, although the associations were relatively limited, we found, in contrast with previous findings, that individuals who place importance on the “time available for cooking” were less likely to be overweight. Indeed, time scarcity has been described as a common barrier for food preparation [3,31,58], leading parents to lean towards relying on various recourses as forms of food choice coping strategies [7,59,60,61,62], such as ready meals, fast foods, or delivery meals, which have been positively associated with overweight [12,13,16]. To explain this discrepancy, additional analyses revealed that participants who place importance on the time available for cooking actually devoted significantly more time to cook. Thus, we can hypothesize that these people are not more affected by time scarcity, but are more concerned about time management: they evaluate the time they have to choose dishes which can be prepared within the time limits. This hypothesis is in accordance with previous results showing that individuals who spend more time in meal preparation are less likely to be overweight [20]. By contrast, our data showed that people who place importance on fatigue were more likely to be overweight. Previous studies highlighted that food preparation requires energy and efforts [3,7,17,28,31,61], while convenience food is considered as a relevant alternative for saving energy [28,59,60,62]. Hence, people experiencing fatigue have been shown to be more likely to use convenience food [7,59,60,61,62], and, thus, to be overweight [12,13,16], supporting our results. Finally, attaching importance to hunger was slightly associated with overweight. Individuals who pay attention to their hunger cues when choosing the dishes to prepare could also be more likely to eat in response to hunger and satiety cues. Such behavior has been previously associated with lower BMI, potentially explaining our results [63].

In our sample, individuals attaching importance on pleasure were more likely to be overweight. Consistent with our results, prior studies in the literature suggested that pleasure from food can lead to non-homeostatic consumption, and therefore to weight gain [64,65,66]. Nonetheless, it has also been suggested that people are overweight because their food choices are overly influenced by hedonism [67], thus raising the issue of the reverse causality. Within this factor, all the items showed a slight positive association with overweight. It is probable that participants who place importance on these criteria when choosing the dishes to be prepared are more likely to invite friends or family to eat. Thus, the positive association with overweight might be due to the fact that people have higher food intakes [68] and consume more food rich in fat [69] when eating occurs in a group setting, especially when the group is composed of familiar people. By contrast, other studies in the literature highlighted that the frequency of family meals was associated with better dietary quality among children and adolescents, as well as a lower weight [23,70,71,72,73], thus suggesting that the presence of the family may positively influence food choice during home meal preparation.

People who placed importance in the specific diets factor were more likely to be overweight. This factor comprised items regarding “dieting to lose weight”, “dieting because of health problems”, and “diets for personal convictions” (e.g., vegetarianism, religious practices) that exhibited differential associations with overweight. The importance attached to “dieting to lose weight” was associated with a higher likelihood of being overweight. Consistently, giving importance to a weight loss diet might be motivated by a greater weight [74]. “Dieting because of health problems” may include diets due to food intolerances (e.g., gluten, lactose) but also reduced salt or reduced sugar diets. Many of these diets are often followed by individuals who suffer from chronic diseases, such as diabetes, cardiovascular diseases, or hypertension, that are associated with overweight [75], potentially explaining the positive association observed in our sample. Finally, and in accordance with our results, vegetarian diets have been previously associated with lower BMI values [76]. However, the little importance attributed to this criterion in our sample, and the strong positive association observed between the “dieting to lose weight” item and overweight, might explain why the factor relative to specific diets exhibited a positive association with overweight.

Finally, the organization factor exhibited no significant association with overweight. Within this motive the “The dish can be prepared beforehand” item showed a slight positive association with overweight. In the literature organization has been suggested as a food skill that may help individuals to prepare healthier meals [77] and therefore to maintain an adequate weight, however there is, to our knowledge, no data available to support this hypothesis.

Although prospective studies are needed to support our data, helping individuals to take into account healthy eating motives during home meal preparation could be a means in which to consider a public health intervention. However, even if people acknowledge the importance of healthy eating [78], in practical situations pertaining to food choice, our findings highlighted that other motives influence food choice and potentially weight status (i.e., pleasure, specific diets). Thus, helping individuals to balance the different factors influencing food choice in order to integrate healthy eating motives may contribute to encourage healthier food choices and prevent overweight. In the literature, constraints such as time scarcity [7,31,60,61,62] and low cooking skills [13,57,79] have been largely described as barriers for balanced choices during home meal preparation. Cooking classes could be a potential lever to improve cooking skills [80], to increase the ability to manage time and budget constraints [81], and, more generally to balance the different motives identified in our study. Finally, the involvement of children and adolescents in food preparation might also be a way to teach them how to cope with all the factors influencing home meal preparation, since frequency of food preparation during adolescence has been shown to predict a better diet quality in young adults [18].

Given the large sample size of the NutriNet-Santé cohort, our study provided a high statistical power. Second, we used a questionnaire including a very large number of potential motives for dish choice during home meal preparation. Another strength is the wide range of sociodemographic and lifestyle characteristics collected through the web-based platform, which allowed us to control potential effects of confounding factors.

The main limitation of our study was its cross-sectional design which made it impossible to assess causal relationships. Dish choice motives may influence food choices [47,48,49,82] and consequently weight status. However, being overweight might also lead to specific food behaviors and motivations. Thus, a potential reverse or bidirectional causality could not be excluded. Next, due to the recruitment occurring on a voluntary basis, the generalizability of the results is questionable. Indeed, participants are potentially more health-conscious and interested in nutritional issues [83]. It is also important to notice that height and weight were self-reported. Thus, incorrect data may have led to misclassification(s). However, an additional study performed on a subsample of the NutriNet-Santé cohort supported the good validity of self-reported anthropometric data. Compared with clinical data, validity was high with intraclass correlation coefficient ranging from 0.94 for height to 0.99 for weight. Thus, BMI classification was correct in 93% of cases [36].

## 5. Conclusions

The present study is the first to provide data on the association between dish choice motives during home meal preparation and overweight. Our results emphasized that giving importance to a healthy diet motive was negatively associated with overweight having the strongest association, whereas pleasure and specific diets exhibited positive associations. By contrast, no relation was found with constraints and organization. In terms of public health policies, the identification of food choice motives during home meal preparation may be helpful to promote healthier cooking practices in prevention programs. In particular, increasing health awareness and developing food skills enabling individuals to take into account healthy eating criteria when choosing the dish to prepare might have a positive impact. Nonetheless, further research focusing on home-meal preparation is needed to explore how and in what extent these food choices are likely to influence dietary quality, and finally, health status. 

## Figures and Tables

**Table 1:** Individual characteristics of participants according to weight status (*n* = 50,003; NutriNet-Santé cohort 2013).

	BMI < 25 kg/m^2^ *n* = 33,687	BMI ≥ 25 kg/m^2^ *n* = 16,316	*p* ^1^
Sex (%)			<0.0001
Women	83.1	71.6	
Men	16.9	28.4	
Age (%)			<0.0001
18–29	13.5	5.3	
30–49	38.1	29.6	
50–64	34.0	40.9	
≥65	14.4	24.2	
Educational level (%)			<0.0001
Up to secondary	31.6	44.3	
Some college	30.7	28.3	
University degree	36.2	25.4	
Missing data	1.6	2.0	
Monthly income per			<0.0001
Household unit (€/CU) ^2^ (%)			
<1200	13.9	15.8	
1200–1799	22.6	26.1	
1800–2699	24.5	25.0	
≥2700	27.2	24.1	
Missing data	11.8	9.0	
Family status (%)			<0.0001
Living alone, without children	17.5	17.5	
Living with a partner, without children	38.0	42.0	
Household, with at least one child	17.4	14.0	
Household, with at least one adolescent	20.9	20.8	
Household with children and adolescents	6.1	5.6	
Dieting in the past year (%)			<0.0001
No	67.0	36.5	
Yes	33.0	63.5	
Physical activity (%)			<0.0001
Low	16.9	20.6	
Moderate	37.1	32.9	
High	29.0	28.1	
Missing data	16.9	18.4	

CU: Consumption Units; BMI: Body Mass Index. ^1^ *p*-values based on chi-square test; ^2^ One CU is attributed for the first adult in the household, 0.5 for other individuals aged 14 or older, and 0.3 for children under 14.

**Table 2:** Mean ratings of the 27 dish choice motives and the five factors emerging from the exploratory factor analysis in overweight and non-overweight participants (*n* = 50,003; NutriNet-Santé study 2013).

When Choosing the Dishes You Are Going to Cook, How Important Are the Following Criteria?	BMI < 25 kg/m^2^ *n* = 33,687	BMI ≥ 25 kg/m^2^ *n* = 16,316	*p* ^1^
Factor 1: Healthy diet	3.96 ± 0.61 ^2^	3.85 ± 0.64	<0.0001
Use of seasonal products	4.26 ± 0.84 ^3^	4.22 ± 0.85	<0.0001
What I and/or my relatives ate during the previous days	3.67 ± 1.01	3.56 ± 1.04	<0.0001
My eating habits and/or that of my relatives	3.87 ± 0.93	3.71 ± 0.96	<0.0001
Nutritional balance of the meal	3.98 ± 0.89	3.86 ± 0.92	<0.0001
Nutritional balance of the dish	4.04 ± 0.83	3.90 ± 0.87	<0.0001
Factor 2: Constraints	3.79 ± 0.55	3.72 ± 0.58	<0.0001
Time available for cooking	3.93 ± 0.98	3.74 ± 1.05	<0.0001
My cooking skills	3.57 ± 1.03	3.56 ± 1.03	0.66
Ingredients at my disposal	4.16 ± 0.80	4.08 ± 0.83	<0.0001
Leftovers in my refrigerator/freezer	3.73 ± 0.96	3.65 ± 0.98	<0.0001
My state of fatigue	3.68 ± 0.99	3.65 ± 1.03	0.0003
My hunger and/or that of my relatives	3.68 ± 0.86	3.63 ± 0.86	<0.0001
Factor 3: Pleasure	3.37 ± 0.58	3.42 ± 0.60	<0.0001
My preferences and/or those of my relatives	4.20 ± 0.71	4.16 ± 0.74	<0.0001
The dish can be adapted to please all guests	3.36 ± 1.17	3.49 ± 1.12	<0.0001
Originality of the dish	2.63 ± 0.98	2.76 ± 1.00	<0.0001
What I and/or my relatives want to eat	3.91 ± 0.80	3.89 ± 0.80	0.031
Recipes I come across	2.76 ± 0.98	2.79 ± 1.06	0.014
Factor 4: Specific diets	2.75 ± 1.02	2.93 ± 0.92	<0.0001
My personal convictions and/or that of my relatives	1.99 ± 1.34	1.85 ± 1.23	<0.0001
My health status and/or that of my relatives	3.39 ± 1.38	3.57 ± 1.26	<0.0001
My eventual diet to lose weight and/or that of my relatives	2.88 ± 1.34	3.38 ± 1.20	<0.0001
Factor 5: Organization	2.88 ± 0.88	2.89 ± 0.86	0.37
What I planned to eat (meal planning)	2.55 ± 1.20	2.56 ± 1.19	0.23
The dish can be prepared beforehand	3.12 ± 1.12	3.14 ± 1.09	0.058
The dish can be prepared in large quantities	2.98 ± 1.18	2.97 ± 1.15	0.29

BMI: Body Mass Index. ^1^ *p*-value based on Student’s *t*-test. ^2^ Means ± SD. Corresponds to the mean of the different criteria loading in the factor. Ranges from 1 (not important at all) to 5 (very important). Higher score reflects greater importance placed on the factor (for all such values); ^3^ Means ± SD. Corresponds to the importance attributed to each criterion on the 5-points Likert scale (not important at all to very important). Higher score reflects greater importance placed on the criteria (for all such values).

**Table 3:** Multivariate logistic regression analyses showing the association between dish choice motives and overweight (including obesity) in weekdays (*n* = 50,003; NutriNet-Santé cohort 2013).

	OR (95% CI)	*p* ^1^
**Factor 1: Healthy diet**	0.65 (0.62; 0.67)	<0.0001
**Factor 2: Constraints**	1.03 (0.99; 1.08)	0.14
**Factor 3: Pleasure**	1.14 (1.10; 1.19)	<0.0001
**Factor 4: Specific diets**	1.19 (1.17; 1.22)	<0.0001
**Factor 5: Organization**	1.01 (0.98; 1.03)	0.62

^1^ Adjusted for other dish choice motives, sex, age, educational level, monthly income, family status, physical activity, and dieting to lose weight in the past year.

**Table 4:** Multivariate logistic regression analyses showing the association between criteria related to dish choice motives and overweight (including obesity) in weekdays (*n* = 50,003; NutriNet-Santé cohort 2013).

	OR (95% CI)	*p* ^1^
Factor 1: Healthy diet		
Use of seasonal products	0.91 (0.89; 0.94)	<0.0001
What I and/or my relatives ate during the previous days	0.92 (0.90; 0.94)	<0.0001
My eating habits and/or that of my relatives	0.80 (0.78; 0.82)	<0.0001
Nutritional balance of the meal	0.82 (0.80; 0.84)	<0.0001
Nutritional balance of the dish	0.77 (0.75; 0.79)	<0.0001
Factor 2: Constraints		
Time available for cooking	0.97 (0.95; 1.00)	0.022
My cooking skills	1.01 (0.99; 1.03)	0.52
Ingredients at my disposal	1.01 (0.98; 1.04)	0.46
Leftovers in my refrigerator/freezer	1.00 (0.98; 1.02)	0.93
My state of fatigue	1.09 (1.06; 1.11)	<0.0001
My hunger and/or that of my relatives	0.97 (0.95; 1.00)	0.041
Factor 3: Pleasure		
My preferences and/or those of my relatives	1.04 (1.01; 1.07)	0.011
The dish can be adapted to please all guests	1.03 (1.01; 1.05)	0.0014
Originality of the dish	1.06 (1.04; 1.08)	<0.0001
What I and/or my relatives want to eat	1.07 (1.04; 1.10)	<0.0001
Recipes I come across	1.04 (1.02; 1.06)	0.0006
Factor 4: Specific diets		
My personal convictions and/or that of my relatives	0.96 (0.94; 0.97)	<0.0001
My health status and/or that of my relatives	1.09 (1.07; 1.11)	<0.0001
My eventual diet to lose weight and/or that of my relatives	1.45 (1.43; 1.48)	<0.0001 ^2^
Factor 5: Organization		
What I planned to eat (meal planning)	0.99 (0.97; 1.01)	0.20
The dish can be prepared beforehand	1.02 (1.00; 1.04)	0.024
The dish can be prepared in large quantities	1.00 (0.98; 1.02)	0.80

^1^ Adjusted for other dish choice motives, sex, age, educational level, monthly income, family status, physical activity, and dieting to lose weight in the past year; ^2^ Adjusted for other dish choice motives, sex, age, educational level, monthly income, family status, and physical activity

## Abbreviations

The following abbreviations are used in this manuscript:

BMI	Body Mass Index
CU	Consumption Units
